# Identification of KMU-3, a Novel Derivative of Gallic Acid, as an Inhibitor of Adipogenesis

**DOI:** 10.1371/journal.pone.0109344

**Published:** 2014-10-06

**Authors:** Yu-Kyoung Park, Jinho Lee, Victor Sukbong Hong, Jong-Soon Choi, Tae-Yoon Lee, Byeong-Churl Jang

**Affiliations:** 1 Department of Molecular Medicine, College of Medicine, Keimyung University, Dalseo-gu, Daegu, Republic of Korea; 2 Department of Chemistry, College of Natural Sciences, Keimyung University, Dalseo-gu, Daegu, Republic of Korea; 3 Division of Life Science, Korea Basic Science Institute, Yuseong-gu, Daejeon, Republic of Korea; 4 Graduate School of Analytical Science and Technology, Chungnam National University, Daejeon, Republic of Korea; 5 Department of Microbiology, College of Medicine, Yeungnam University, Nam-gu, Daegu, Korea; National University of Singapore, Singapore

## Abstract

Differentiation of preadipocyte, also called adipogenesis, leads to the phenotype of mature adipocyte. Excessive adipogenesis, however, is largely linked to the development of obesity. Herein we investigated a library of 53 novel chemicals, generated from a number of polyphenolic natural compounds, on adipogenesis. Strikingly, among the chemicals tested, KMU-3, a derivative of gallic acid, strongly suppressed lipid accumulation during the differentiation of 3T3-L1 preadipocytes into adipocytes. On mechanistic levels, KMU-3 inhibited expressions of CCAAT/enhancer-binding protein-α (C/EBP-α), peroxisome proliferator-activated receptor-γ (PPAR-γ), and fatty acid synthase (FAS) during adipocyte differentiation. Moreover, KMU-3 reduced expressions of adipokines, including retinol binding protein-4 (RBP-4), leptin, and regulated on activation, normal T cell expressed and secreted (RANTES) during adipocyte differentiation. Of further note, KMU-3 rapidly blocked the phosphorylation of signal transducer and activator of transcription-3 (STAT-3) during the early stage of adipogenesis. Importantly, pharmacological inhibition studies revealed that AG490, a JAK-2/STAT-3 inhibitor suppressed adipogenesis and STAT-3 phosphorylation, implying that early blockage of STAT-3 activity is crucial for the KMU-3-mediated anti-adipogenesis. These findings demonstrate firstly that KMU-3 inhibits adipogenesis by down-regulating STAT-3, PPAR-γ, C/EBP-α, and FAS. This work shows that KMU-3 is an inhibitor of adipogenesis and thus may have therapeutic potential against obesity.

## Introduction

Obesity is a high risk factor for the development of many human pathologies, including insulin resistance, type 2 diabetes, hyperlipidemia, hypertension, cardiovascular disease, and cancer [1], [2]. Mounting evidence suggests that obesity is a multi-factorial disorder caused by a variety of genetic and endocrine abnormalities, some medicines, a low metabolic rate, nutritional and environmental factors, as well as imbalance of energy homeostasis [3],[4]. A number of studies have demonstrated that adipose tissue plays a critical role in the regulation of energy metabolism by secreting adipokines [4]–[6]. However, there is strong evidence suggesting that abnormal expansion/accumulation of adipose tissue, which is largely associated with excessive adipocyte differentiation, increased numbers (hyperplasia) and lipid contents (hypertrophy) of fat cells, are closely linked to the development of obesity [3], [7], [8]. Thus, any compound that inhibits excessive adipocyte differentiation and/or adipocyte hyperplasia/hypertrophy may have preventive and/or therapeutic potential against obesity and obesity-related disease.

Research has accumulated to indicate that the differentiation of preadipocyte into adipocyte is controlled by a complex network of a variety of cellular proteins, including transcription factors, cell cycle-related proteins, adipocyte-specific genes, lipogenic enzymes, and signaling proteins. For instance, it has been shown that the family of CCAAT-enhancer binding proteins (C/EBP-α, -β and -δ) and peroxisome proliferator-activated receptors (PPAR-γ, -α and -β) plays critical roles in adipocyte differentiation [9]–[11]. Moreover, there is recent evidence suggesting that the janus-activated protein kinase-2 (JAK-2)/signal transducer and activator of transcription-3 (STAT-3) signaling complexes are involved in adipocyte differentiation, particularly at the early stage of adipogenesis [12]. A number of signaling proteins and factors, including adenosine 3′,5′-cyclic monophosphate (cAMP), protein kinase Cs (PKCs), and extracellular-signal regulated protein kinase-1/2 (ERK-1/2), have also shown to have a role in controlling adipocyte differentiation [13]–[16].

3T3-L1 is a well-established murine preadipocyte cell line [17] and has widely been used for understanding the molecular regulation of adipocyte differentiation and for screening potential anti-obesity drugs or agents. Interestingly, recent studies have shown that a number of polyphenolic natural products inhibit adipogenesis [18], [19] and have anti-proliferative and/or pro-apoptotic effects on 3T3-L1 adipocytes at high concentrations [18]–[21]. However, it is presumed that anti-adipogenic activity of polyphenolic natural compounds may be limited by their physicochemical properties such as high hydrophilicity and/or cell toxicity at high concentrations. We have recently used diversity-oriented synthesis to construct a library that consists of 53 novel chemicals based on several natural products, including gallic acid (GA). In this study, we investigated the effect of individual 53 chemicals on lipid accumulation during the differentiation of 3T3-L1 preadipocytes into adipocytes. Here we report for the first time that among 53 chemicals screened, N-(4-(*tert*-Butyl)phenyl)-3,4,5-trihydroxybenzamide (KMU-3), a novel derivative of GA, strongly inhibits adipogenesis without inducing cell toxicity. Our data also show that the KMU-3-mediated anti-adipogenic effect is closely linked to down-regulation of PPAR-γ, C/EBP-α, and STAT-3.

## Materials and Methods

### Materials

Polyclonal C/EBP-α antibody, monoclonal C/EBP-β antibody, monoclonal PPAR-γ antibody, monoclonal STAT-3 antibody, monoclonal phospho-STAT-3 (p-STAT-3) antibody, polyclonal STAT-1 antibody, monoclonal p-STAT-1 antibody, polyclonal STAT-5 antibody, polyclonal p-STAT-5 antibody, polyclonal JAK-2 antibody, polyclonal p-JAK-2 and polyclonal Src-homology 2 (SH2) domain phosphatase-1 (SHP-1) antibody were purchased from Santa Cruz Biotechnology (Santa Cruz, CA). Polyclonal JAK-1 antibody, polyclonal p-JAK-1 antibody, polyclonal Src antibody, polyclonal p-Src antibody, polyclonal ERK-1/2 antibody and polyclonal p-ERK-1/2 antibody were purchased from Cell Signaling (Danvers, MA). Monoclonal β-actin (actin) antibody, gallic acid and AG490 were purchased from Sigma (St. Louis, MO).

### Synthesis of N-(4-(tert-Butyl)phenyl)-3,4,5-trihydroxybenzamide (KMU-3)

To a 10 mL *N,N*-dimethylformamide solution of gallic acid (0.20 g, 1.1 mmol) were added 1-hydroxybenzotriazole (0.28 g(2.12 mmol), 1-ethyl-3-(3-dimethylaminopropyl)carbodiimide (0.19 g(1.59 mmol), and 4-*tert*-butylaniline (0.96 mL, 8.1 mmol). After stirring the reaction mixture for 2 h at RT, solvent was removed under reduced pressure. The residue was dissolved in ethyl acetate and was washed with saturated aqueous sodium bicarbonate solution and 5% citric acid solution, water, and brine, sequentially. The organic layer was dried using magnesium sulfate and filtered. The filtrate was concentrated under reduced pressure and purified by flash column chromatography using hexane∶ ethyl acetate∶ methanol (7∶7∶1) mixture as eluent to afford the product 0.070 g in 28% yield; ^1^H NMR (CDCl_3_+CD_3_OD, 400 MHz) δ 7.54 (d, *J* = 8.4 Hz, 2H), 7.37 (d, *J* = 8.8 Hz, 2H), 6.96 (s, 2H), 1.32 (s, 9H); ^13^C NMR (CDCl_3_+CD_3_OD, 100 MHz) δ 171.05, 151.26, 148.85, 140.58, 139.47, 129.52, 129.33, 124.47, 110.80, 38.20, 35.11; ESI-MS: m/z 302 (M+H)^+^, 324 (M+Na)^+^. Other chemicals used in this study were also prepared in similar ways to the preparation of KMU-3, except for the parent compound. KMU-3 (and other chemicals) was dissolved in 100% DMSO and prepared as 10 mM stock solution.

### Cell culture and differentiation

3T3-L1 preadipocytes (ATCC, Manassas, VA) were grown up to the contact inhibition stage and remained in the post-confluent stage for 2 days in Dulbecco's modified Eagle's medium (DMEM) supplemented with 10% calf bovine serum (Gibco, Grand Island, NY) and penicillin–streptomycin (Welgene, Daegu). Differentiation was then induced by changing the medium to DMEM supplemented with 10% fetal bovine serum (FBS) (Welgene, Daegu) plus a cocktail of hormones (MDI) that include 0.5 mM 3-isobutyl-1-methylaxanthine (M) (Sigma), 0.5 µM dexamethasone (D) (Sigma), and 5 µg/ml insulin (I) (Sigma) in the presence or absence of KMU-3 at the indicated concentrations. After 48-h (day 2) MDI-induction, the differentiation medium was replaced with DMEM supplemented with 10% FBS and 5 µg/ml insulin in the presence or absence of KMU-3 at the indicated concentrations. The cells were then fed every other day with DMEM containing 10% FBS in the presence or absence of KMU-3 at the indicated concentrations until day 8. On day 8, the preadipocytes became mature adipocytes that rounded-up and filled with many oil droplets.

### Cell viability assay

3T3-L1 preadipcoytes were seeded into 96-well plates and incubated overnight. Cells were then treated with or without individual 53 chemicals at the indicated concentrations for 24 h, followed by incubating with MTS (3-(4,5-dimethylthiazol-2-yl)-5-(3-carboxymethoxy phenyl)-2-(4-sulfophenyl)-2H-tetrazolium) reagent (Promega, Madison, WI) for 1.5 h at 37°C. The absorbance was measured at 490 nm using a microplate reader (Bio-Rad, Hercules, CA).

### Oil red O staining

On day 8 of differentiation, 3T3-L1 cells were washed twice with PBS, fixed with 10% formaldehyde for 2 h at room temperature, washed with 60% isopropanol and dried completely. The fixed cells were then stained with Oil red O working solution that was prepared by diluting 0.5% stock solution in 99% isopropanol with distilled water (6∶4 ratio) for 1 h at room temperature (RT) and then washed twice with distilled water. Lipid droplets were observed by light microscopy (Nikon).

### Quantification of intracellular triglyceride (TG) content by AdipoRed assay

After 8 days of treatment, lipid content was measured using a commercially available AdipoRed Assay Reagent kit according to the manufacturer's instructions (Lonza). After a 10-min incubation, the plates were placed in a Victor^3^ (Perkin Elmer), and fluorescence was measured with an excitation wavelength of 485 nm and an emission wavelength of 572 nm.

### Preparation of whole cell lysates

At the designated time point, 3T3-L1 cells were washed twice with PBS and exposed to a modified RIPA buffer [50 mM Tris-Cl (pH 7.4), 150 mM NaCl, 0.1% sodium dodecyl sulfate, 0.25% sodium deoxycholate, 1% Triton X-100, 1% Nonidet P-40, 1 mM EDTA, 1 mM EGTA, proteinase inhibitor cocktail (1x)]. The cell lysates were collected in a 1.5 mL tube and centrifuged at 12,000 rpm for 20 min at 4°C. The supernatant was saved and protein concentrations were determined with Bradford reagent (Bio-Rad).

### Western blot analysis

Proteins (50 µg) were separated by SDS-PAGE (10%) and transferred onto nitrocellulose membranes (Millipore). The membranes were washed with TBS (10 mM Tris, 150 mM NaCl) supplemented with 0.05% (vol/vol) Tween 20 (TBST) followed by blocking with TBST containing 5% (wt/vol) non-fat dried milk. The membranes were incubated overnight with antibodies specific for C/EBP-α (1∶1,000), C/EBP-β (1∶1,000), PPAR-γ (1∶1,000), STAT-3 (1∶1,000), p-STAT-3 (1∶1,000), STAT-1 (1∶1,000), p-STAT-1 (1∶1,000), STAT-5 (1∶1,000), p-STAT-5 (1∶1,000), JAK-2 (1∶1,000), p-JAK-2 (1∶1,000), SHP-1 (1∶1,000), JAK-1 (1∶2,000), p-JAK-1 (1∶2,000), Src (1∶2,000), p-Src (1∶2,000), ERK-1/2 (1∶2,000), p-ERK-1/2 (1∶2,000) or β-actin (1∶10,000) at 4°C. The membranes were then exposed to secondary antibodies coupled to horseradish peroxidase for 2 h at RT. The membranes were washed three times with TBST at RT. Immunoreactivities were detected by ECL reagents. Equal protein loading was assessed by the expression level of actin protein.

### Reverse transcription-polymerase chain reaction (RT-PCR)

At the designated time point, total cellular RNA from the control or KMU-3-treated 3T3-L1 cells was isolated with the RNAzol-B (Tel-Test, Friendswood, TX). Three micrograms of total RNA were reverse transcribed using a random hexadeoxynucleotide primer and reverse transcriptase. Single stranded cDNA was amplified by PCR with the following primers. Primer sequences used for amplifications were as follows: C/EBP-α sense 5′-TTACAACAGGCCAGGTTTCC-3′; C/EBP-α antisense 5′-CTCTGGGATGGATCGATTGT-3′; C/EBP-β sense 5′-CGAGCGCAACAACATCGCGG-3′; C/EBP-β antisense 5′-TACTCAGGGCCCGGCTGACA-3′; PPAR-γ sense 5′-GGTGAAACTCTGGGAGATTC-3′; PPAR-γ antisense 5′-CAACCATTGGGTCAGCTCTC-3′; fatty acid synthase (FAS) sense 5′-TTGCTGGCACTACAGAATGC-3′; FAS antisense 5′-AACAGCCTCAGAGCGACAAT-3′; fatty acid binding protein (aP2) sense 5′- AGTGGGAGTGGGCTTTGCCA-3′; aP2 antisense 5′-GGGCCCCGCCATCTAGGGTTA-3′; Adiponectin sense 5′-GGAGATGCAGGTCTTCTTGGT-3′; Adiponectin antisense 5′- TCCTGATACTGGTCGTAGGTGAA-3′; Leptin sense 5′-CCAAAACCCTCATCAAGACC-3′; Leptin antisense 5′-CTCAAAGCCACCACCTCTGT-3′; retinol binding protein-4 (RBP4) sense 5′- ACTGGGGTGTAGCCTCCTTT-3′; RBP4 antisense 5′-GGTGTCGTAGTCCGTGTCG-3′; regulated on activation, normal T cell expressed and secreted (RANTES) sense 5′-TCCAATCTTGCAGTCGTGTTTG-3′; RANTES antisense 5′-TCTGGGTTGGCACACACTTG-3′; Actin sense 5′-GGTGAAGGTCGGTGTGAACG-3′; Actin antisense 5′-GGTAGGAACACGGAAGGCCA-3′. The PCR conditions applied were: C/EBP-α, 30 cycles of denaturation at 95°C for 30 s, annealing at 62°C for 30 s, and extension at 72°C for 30 s; C/EBP-β, 30 cycles of denaturation at 95°C for 30 s, annealing at 53°C for 30 s, and extension at 72°C for 30 s; PPAR-γ, 30 cycles of denaturation at 95°C for 30 s, annealing at 53°C for 30 s, and extension at 72°C for 30 s; FAS, 30 cycles of denaturation at 95°C for 15 s, annealing at 55°C for 40 s, and extension at 68°C for 45 s; aP2, 30 cycles of denaturation at 95°C for 30 s, annealing at 59°C for 30 s, and extension at 72°C for 30 s; Adiponectin, 30 cycles of denaturation at 95°C for 1 min, annealing at 53°C for 1 min, and extension at 72°C for 1 min; Leptin, 30 cycles of denaturation at 95°C for 1 min, annealing at 57°C for 1 min, and extension at 72°C for 1 min; RBP4, 35 cycles of denaturation at 95°C for 1 min, annealing at 53°C for 1 min, and extension at 72°C for 1 min; RANTES, 35 cycles of denaturation at 95°C for 1 min, annealing at 56°C for 1 min, and extension at 72°C for 1 min; Actin, 25 cycles of denaturation at 95°C for 30 s, annealing at 57°C for 30 s, and extension at 72°C for 1 min. Expression levels of actin mRNA were used as an internal control to evaluate the relative mRNA expression of adipocyte-specific genes and adipokines.

### Statistical analyses

MTS analysis was done in triplicates and repeated three times. Data were expressed as mean ± standard error (SE). The significance of difference was determined by One-Way ANOVA. All significance testing was based on upon a *p* value of <0.05.

## Results

### Individual 53 novel chemicals demonstrate no cytotoxic activity against 3T3-L1 preadipocytes

**Figure 1:**
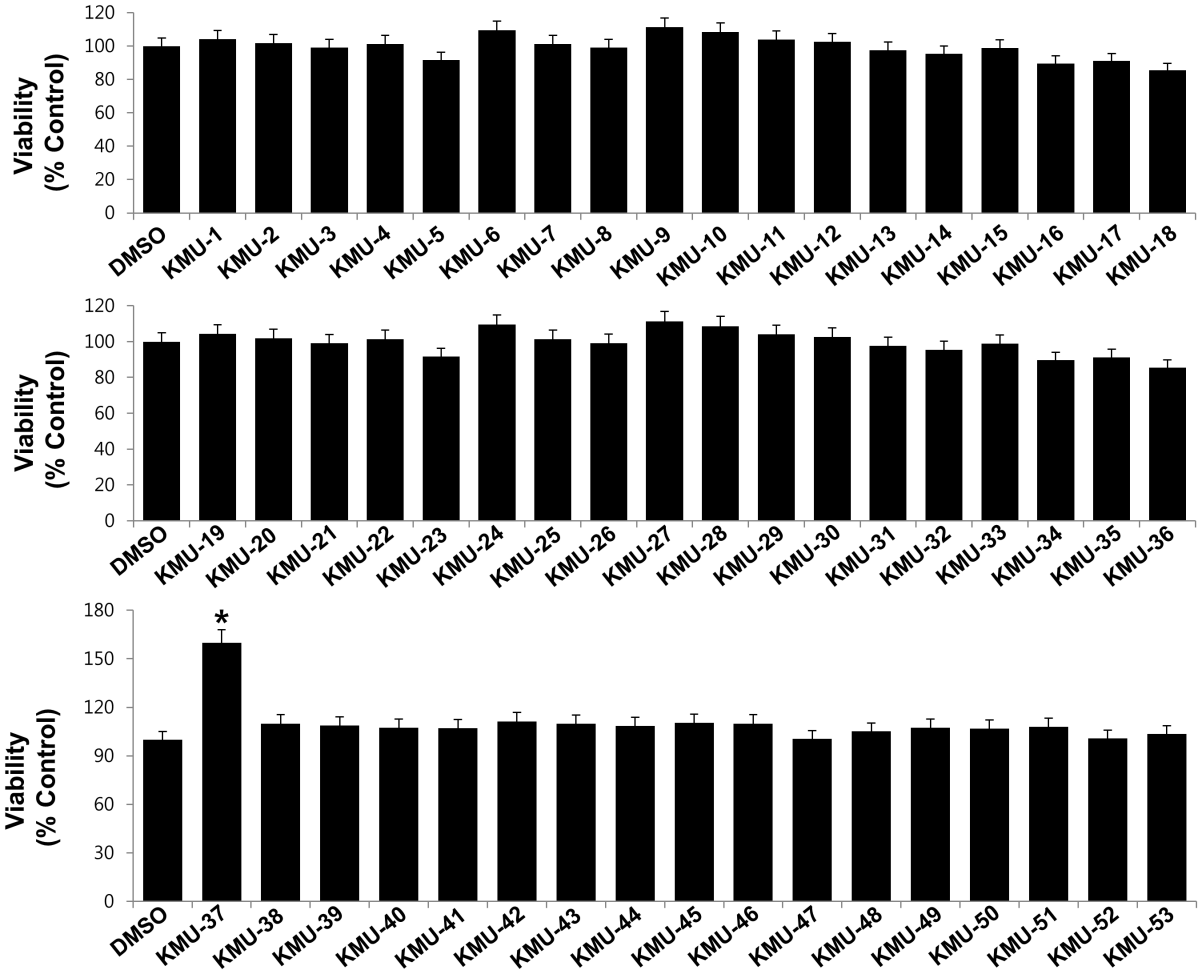
Effects of 53 novel chemicals on the viability of 3T3-L1 preadipocytes. 3T3-L1 preadipocytes were treated with or without each of the test compounds at a final concentration of 10 µM for 24 h. The cell viability was measured by MTS assay. Data are mean ± SE from three independent experiments with three replicates. *P<0.05 vs. control (no chemical).

The goal of this study was to identify compounds that can inhibit adipogenesis in 3T3-L1 cells. Initially, to see whether the test compounds were cytotoxic, the preadipocytes were treated with or without each of the 53 chemicals at 10 µM concentration for 24 h, followed by measurement of any change of the cell viability using a MTS assay. As shown in Fig. 1, no cytotoxicity to 3T3-L1 preadipocytes was noted for these compounds; rather, one of the compounds, KMU-37, substantially enhanced the cell viability.

### KMU-3, a novel synthetic derivative of gallic acid, has strong anti-adipogenic effects

**Figure 2:**
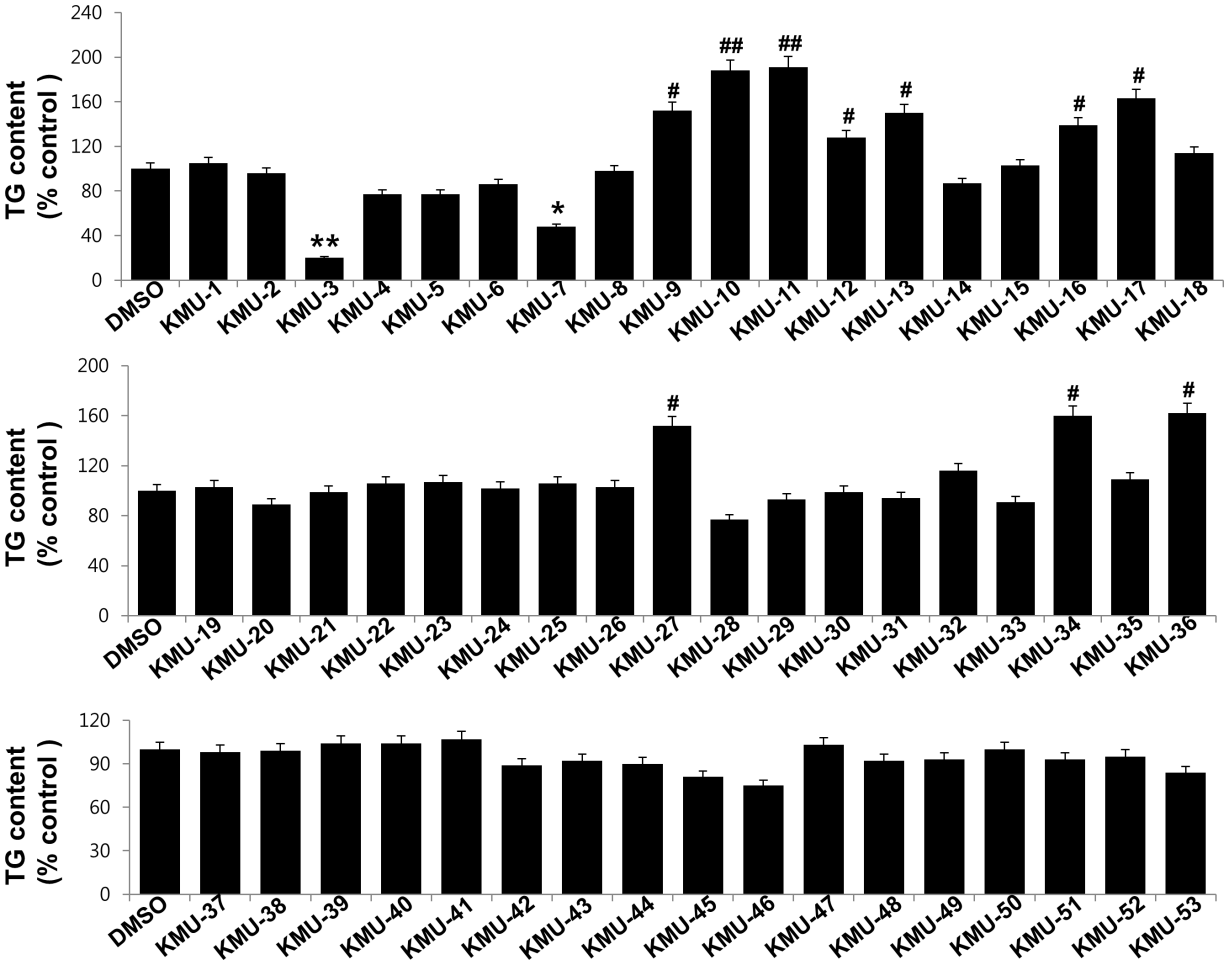
Effects of 53 novel chemicals on intracellular triglyceride (TG) synthesis during 3T3-L1 adipocyte differentiation. 3T3-L1 preadipocytes were induced to differentiate with induction medium containing MDI (M, IBMX (0.5 mM); D, dexamethasone (0.5 µM); I, insulin (5 µg/ml)), insulin (5 µg/ml), and FBS (10%) in the presence or absence of 10 µM of the test compounds for 8 days. On day 8, the cellular TG contents were quantified by AdipoRed assay. Values are mean ± SE of data from three independent experiments with three replicates. *P or ^#^P<0.05 and **P or ^##^P<0.01 vs. control (no chemical).

**Figure 3:**
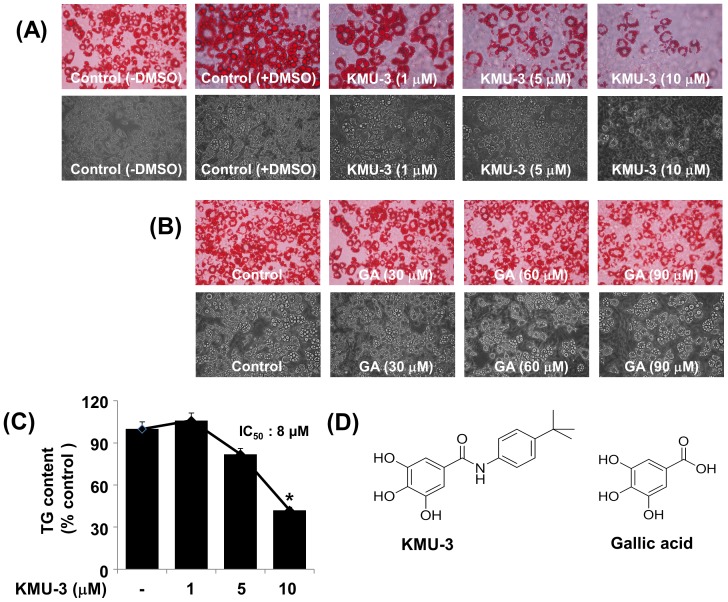
Effect of KMU-3, a novel synthetic derivative of gallic acid, on lipid accumulation during 3T3-L1 adipocyte differentiation. (A,B) 3T3-L1 preadipocytes were induced to differentiate with induction medium containing MDI, insulin, and FBS in the presence or absence of KMU-3 (A) or gallic acid (GA) (B) at the indicated concentrations for 8 days. On day 8, the cellular lipid contents were assessed by Oil Red O staining. Phase-contrast images of the cells were also taken after the treatment (lower panels in A or B). Each picture in (A) and (B) is a representative of three independent experiments. (C) 3T3-L1 preadipocytes were induced to differentiate with induction medium containing MDI, insulin, and FBS in the presence or absence of KMU-3 at the indicated concentrations for 8 days. On day 8, the cellular TG contents were quantified by AdipoRed assay. Values are mean ± SE of data from three independent experiments with three replicates. *P<0.05 vs. control (no chemical). (D) The chemical structure of KMU-3 and GA.

We next investigated the effect of individual chemicals on lipid accumulation at a final concentration of 10 µM during adipocyte differentiation using an AdipoRed assay. There were high triglyceride (TG) contents in 3T3-L1 preadipocytes incubated with induction medium containing adipogenic cocktails and inducers (MDI, insulin, and FBS) on day 8 of cell differentiation (Fig. 2). Cells treated with KMU-3, a derivative of gallic acid (GA), however, exhibited a striking reduction in levels of TG contents, compared with the mock-treated cells. KMU-7 also substantially reduced TG contents. On the contrary, treatment with several chemicals, including KMU-10 and KMU-11, enhanced TG contents in the preadipocytes that were co-incubated with induction medium, suggesting their pro-adipogenic effects. Treatment with all other chemicals did not largely affect TG contents under these experimental conditions. Because KMU-3 showed a very powerful inhibitory effect on TG synthesis, we selected it for further studies. The lipid-reducing effect of KMU-3 with different concentrations (1, 5, and 10 µM) was next determined by an Oil Red O staining. As shown in Fig. 3A, many lipid droplets were formed in 3T3-L1 preadipocytes in induction medium only on day 8 of cell differentiation. However, treatment of the preadipocytes with KMU-3 strikingly reduced the amounts of lipid droplets in a dose-dependent manner. Apparently, KMU-3 at the dose of 10 µM maximally reduced lipid droplets. Interestingly, treatment with its parent compound, gallic acid (GA) at 30, 60 or 90 µM did not affect the formation of lipid droplets in the preadipocytes when co-incubated with induction medium. The lipid-reducing effect of KMU-3, but not GA, was also observed by light microscopic measurement (Fig. 3A and B, lower panels). Data of AdipoRed assay further revealed a dose-dependent reduction of TG contents by KMU-3 with the IC_50_ of about 8 µM (Fig. 3C). The chemical structure of KMU-3 and GA is shown in Fig. 3D, respectively. Because of strong inhibitory effects on both lipid accumulation and TG synthesis, we selected the concentration of 10 µM of KMU-3 for further studies.

### KMU-3 differentially inhibits the expression of C/EBP-α and PPAR-γ and the phosphorylation of STAT-3 during adipocyte differentiation

**Figure 4:**
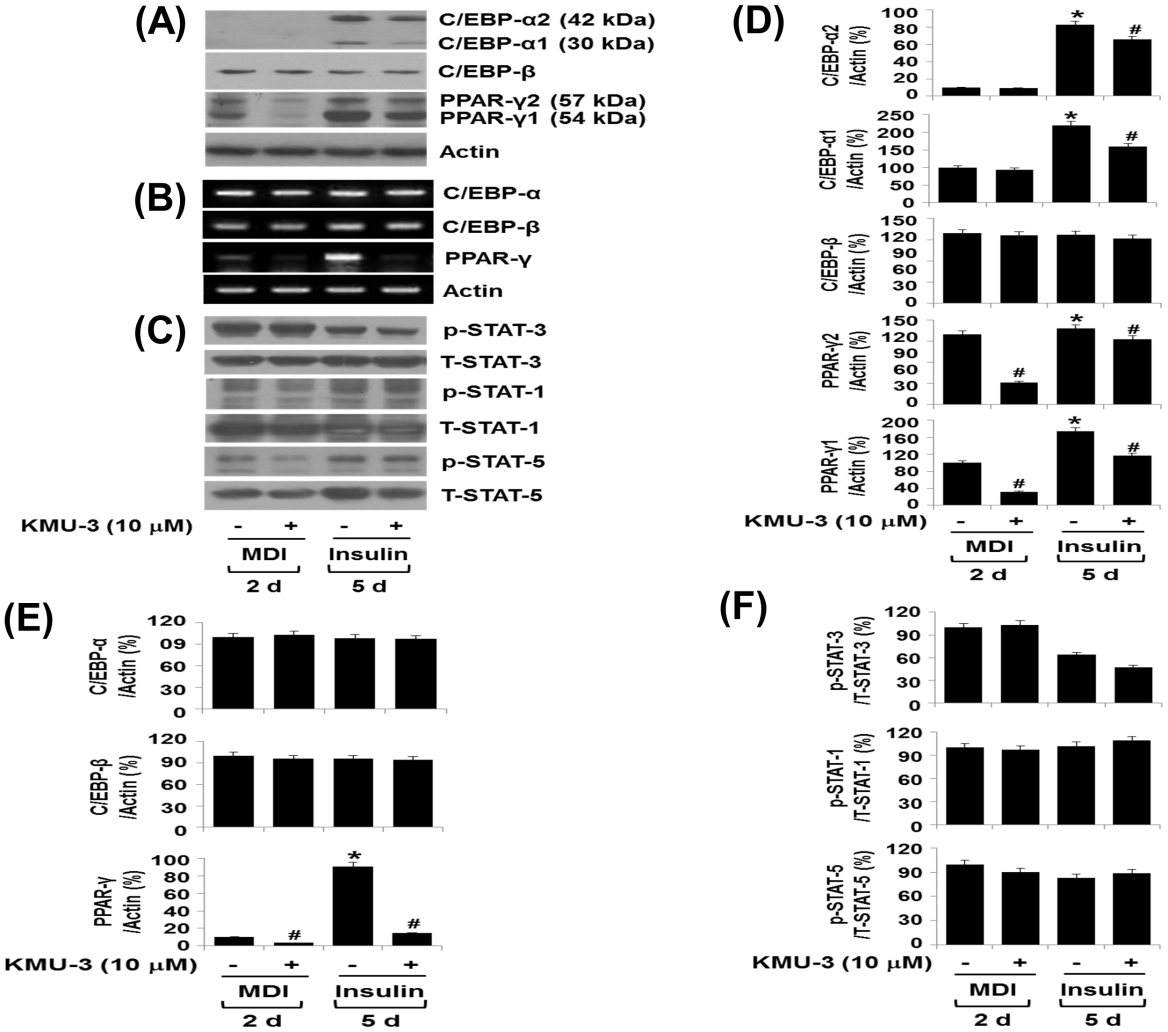
Effect of KMU-3 on expressions and/or activities of C/EBP-α, C/EBP-β, PPAR-γ, and STATs during 3T3-L1 adipocyte differentiation. (A,B,C) 3T3-L1 preadipocytes were induced to differentiate with induction medium containing MDI and insulin in the presence or absence of KMU-3 (10 µM) and harvested at day 2 and 5, respectively. The cellular protein and mRNA at the indicated time point were extracted and analyzed by Western blot (A, C) and RT-PCR (B) analysis, respectively. Each picture in (A), (B), and (C) is a representative of three independent experiments. (D) and (E) The densitometry data of (A) and (B), respectively, that show C/EBP-α, C/EBP-β, and PPAR-γ protein and mRNA levels normalized to actin protein and mRNA levels as percentage of the value at 0 day. (F) The densitometry result of (C) that shows p-STAT-3, p-STAT-1 and p-STAT-5 protein levels normalized to total expression levels of each protein as percentage of the value at 0 day. *p<0.05 compared to the value of day 0. ^#^p<0.05 compared to the value of KMU-3 free control at the indicated days.

**Figure 5:**
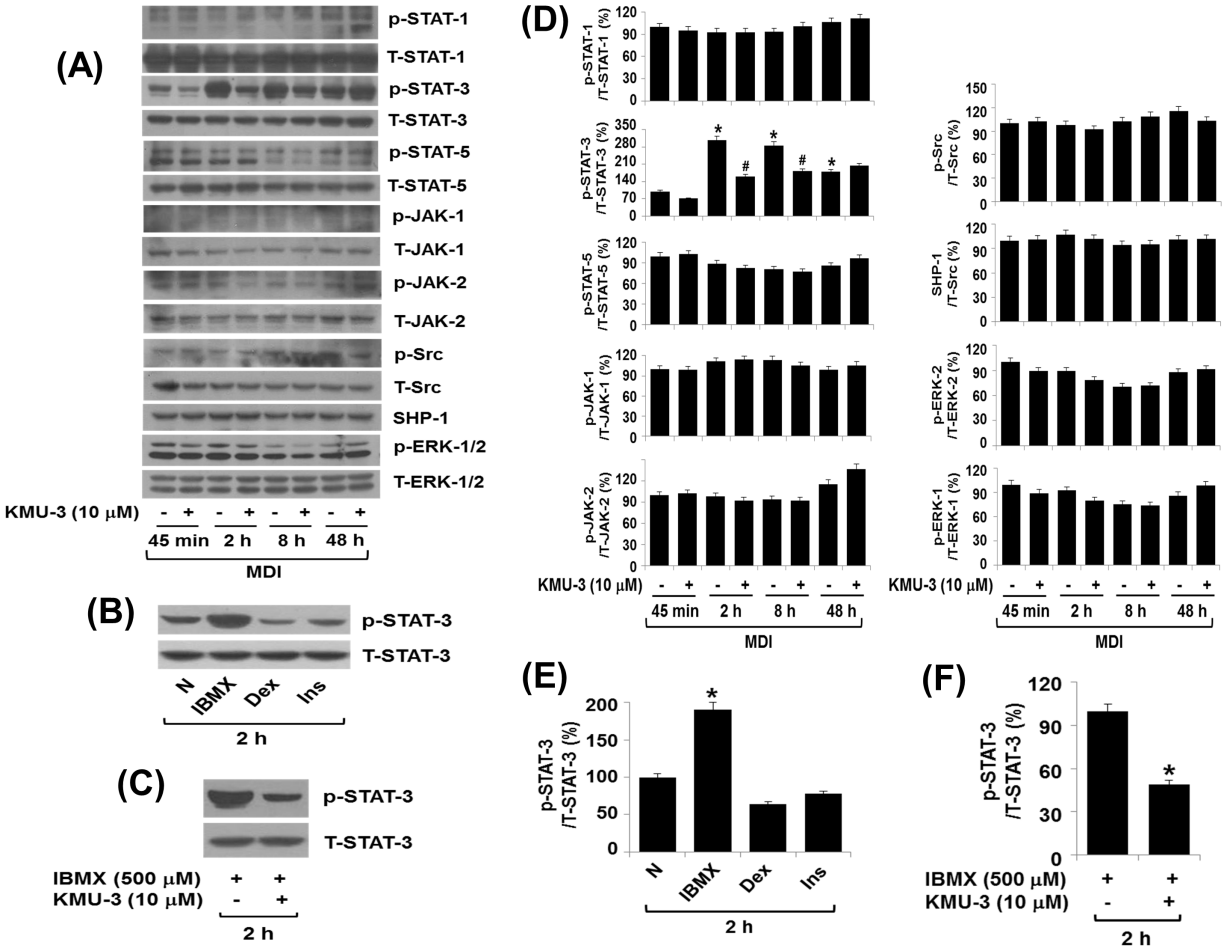
Effect of KMU-3 on expressions and/or activities of STATs, JAKs, Src, SHP-1, and ERK-1/2 in 3T3-L1 preadipocytes incubated with MDI and/or IBMX. (A) 3T3-L1 preadipocytes were treated with induction medium containing MDI in the presence or absence of KMU-3 (10 µM) and harvested at the time of 45 min, 2, 8, and 48 h, respectively. The cellular protein at the indicated time point was extracted and analyzed by Western blot analysis. (B) 3T3-L1 preadipocytes were treated without (N) or with IBMX (0.5 mM), dexamethasone (Dex, 0.5 µM), and insulin (Ins, 5 µg/ml), respectively, for 2 h. The cellular protein was then extracted and analyzed by Western blot analysis. (C) 3T3-L1 preadipocytes were treated with IBMX (0.5 mM) in the presence or absence of KMU-3 (10 µM) for 2 h. The cellular protein was then extracted and analyzed by Western blot analysis. Each picture in (A), (B), and (C) is a representative of three independent experiments. (D) The densitometry result of (A) that shows p-STAT-1, p-STAT-3, p-STAT-5, p-JAK-1, p-JAK-2, p-Src, SHP-1, p-ERK-1, and p-ERK-2 protein levels normalized to total expression levels of STAT-1, STAT-3, STAT-5, JAK-1, JAK-2, Src, ERK-1 or ERK-2 protein as percentage of the value at the time 0. (E) and (F) The densitometry data of (B) and (C), respectively, that show p-STAT-3 protein levels normalized to total expression levels of STAT-3 protein as percentage of the value at the same time. *p<0.05 compared to the value at the time 0. ^#^p<0.05 compared to the value of KMU-3 free control at the indicated times.

To understand molecular and cellular mechanisms underlying the KMU-3-mediated anti-adipogenic effect, we next examined the effect of KMU-3 (10 µM) on expression and/or activity (phosphorylation) of the family of C/EBPs, PPARs, and STATs involved in adipogenesis. Western blot analysis revealed a time-dependent increase in the protein levels of C/EBP-α, C/EBP-β, and PPAR-γ in 3T3-L1 preadipocytes produced by treatment with induction medium containing MDI and insulin on day 2 and 5, respectively (Fig. 4A). Importantly, KMU-3 inhibited the adipogenesis-dependent protein expressions of C/EBP-α and PPAR-γ, while not affecting the expression of C/EBP-β. Results of RT-PCR analysis, as shown in Fig. 4B, also demonstrated a time-dependent increase in the mRNA expressions of C/EBP-α, C/EBP-β, and PPAR-γ during adipocyte differentiation. However, of note, KMU-3 inhibited the mRNA expression of PPAR-γ, but did not affect that of C/EBP-α and C/EBP-β during adipocyte differentiation. These results suggest that KMU-3 down-regulates PPAR-γ and C/EBP-α at the transcriptional and post-transcriptional (or translational) level, respectively. Control actin protein and mRNA expressions remained constant under these experimental conditions (Fig. 4A and B). As shown in Fig. 4C, there were higher phosphorylation levels of STAT-3 than those of STAT-1 and STAT-5 in 3T3-L1 preadipocytes produced by differentiation with induction medium containing MDI and insulin on day 2 and 5, respectively. However, KMU-3 had no or little effect on the phosphorylation levels and total protein levels of STAT-1, STAT-3, and STAT-5 during adipocyte differentiation at the designated time points. The densitometry data of Fig. 4A, 4B, and 4C are shown in Fig. 4D, 4E, and 4F, respectively. We next investigated which signal transduction protein(s) is activated/expressed during the early stage of adipogenesis induced by MDI at the treatment time of 45 min, 2, 8 and 48 h, respectively, and if any, KMU-3 inhibits it. As shown in Fig. 5A, strikingly, there was a time-dependent increase in STAT-3 phosphorylation particularly with maximal increase at 2 h, but the MDI-induced STAT-3 phosphorylation was repressed by KMU-3. Although other signaling proteins, including STAT-1, STAT-5, JAK-1, JAK2, Src, SHP-1, and ERK-1/2, were also substantially phosphorylated, their phosphorylation levels were not largely affected in the preadipocytes treated with MDI in the presence or absence of KMU-3 at the times tested. Total protein levels of these signaling proteins including STAT-3 also remained constant under these experimental conditions. We next tested which component(s) among MDI mediates STAT-3 phosphorylation in 3T3-L1 preadipocytes and KMU-3 inhibits the process. Treatment with IBMX, but not dexamethasone or insulin, induced strong STAT-3 phosphorylation and the IBMX-induced STAT-3 phosphorylation was largely blocked by KMU-3 (Fig. 5B and C). The densitometry data of Fig. 5A, 5B, and 5C are shown in Fig. 5D, 5E, and 5F, respectively.

### Inhibition of the early adipogenesis process and phosphorylation of STAT-3 is critical for the KMU-3-mediated anti-adipogenesis

**Figure 6:**
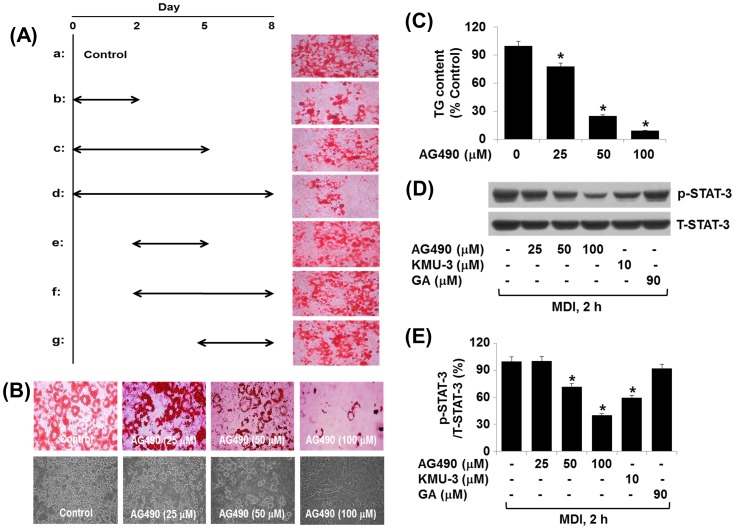
Effect of KMU-3 or AG490 on lipid accumulation and/or STAT-3 phosphorylation during 3T3-L1 adipocyte differentiation. (A) 3T3-L1 preadipocytes were induced to differentiate with induction medium containing MDI, insulin, and FBS in the presence or absence of KMU-3 as described in Materials and Methods (a to g) at the indicated time points (2, 5, 8 days). The cellular lipid contents at each time point were assessed by Oil Red O staining. (B,C) 3T3-L1 preadipocytes were induced to differentiate with induction medium containing MDI, insulin, and FBS in the presence or absence of AG490, a JAK-2/STAT-3 inhibitor, at the indicated concentrations for 8 days. On day 8, cellular lipid accumulation and triglyceride (TG) contents were assessed by Oil Red O staining (B) and AdipoRed assay (C), respectively. Phase-contrast images of the cells were also taken after the treatment (lower panel in B). (D) 3T3-L1 preadipocytes were treated without or with MDI in the presence or absence of AG490 at the indicated concentrations for 2 h. The cellular protein was then extracted and analyzed by Western blot analysis. (E) The densitometry result of (D) in which phosphorylation levels of STAT-3 were quantified with total expression levels of STAT-3. *p<0.05 compared to the value obtained at the same time point (2 h) in the absence of any drug.

Considering the inhibitory effect of KMU-3 on the MDI-induced STAT-3 phosphorylation (Fig. 5A), we further determined whether KMU-3 inhibits the early adipogenesis process. To this end, as depicted in detail in Fig. 6A, 3T3-L1 preadipocytes were incubated with induction medium (MDI, insulin, and FBS) in the presence or absence of KMU-3 (10 µM) at the designated time points. As expected, the mock-treated cells incubated with MDI, insulin, and FBS on day 2, 5, and 8, respectively, had high lipid accumulation (a). However, 3T3-L1 preadipocytes that were initially incubated with MDI plus KMU-3 on day 2 and then treated with insulin and FBS in the absence of KMU-3 on day 5 and 8, respectively, had less lipid contents (b) than (a). Moreover, 3T3-L1 preadipocytes initially incubated with MDI and insulin in the presence of KMU-3 on day 2 and 5, respectively, and then treated with FBS in the absence of KMU-3 on day 8 also had less lipid contents (c) than (a). Also, there were less lipid contents in the preadipocytes incubated, in the presence of KMU-3, with MDI, insulin and FBS on day 2, 5, and 8 (d) than (a). However, 3T3-L1 cells initially incubated with MDI only on day 2, treated with insulin plus KMU-3 on day 5, and grown with FBS only on day 8 had high lipid contents (e). Furthermore, the preadipocytes initially incubated with MDI only on day 2 and then treated with insulin and FBS in the presence of KMU-3 on day 5 and 8, respectively, had high lipid contents (f). Lastly, 3T3-L1 cells initially incubated with MDI and insulin in the absence of KMU-3 on day 2 and 5, respectively, and then treated with FBS plus KMU-3 on day 8 still had high lipid contents (g). These results collectively suggest that KMU-3 inhibits adipogenesis probably in the early phases. Using AG490, an inhibitor of JAK-2/STAT-3, we next confirmed the role of STAT-3 inhibition in the KMU-3-mediated anti-adipogenesis. Compared with the mock-treated cells, cells treated with AG490 exhibited a reduction in lipid accumulation in a concentration-dependent manner in which the maximal inhibition was seen at 100 µM. The lipid-reducing effect of AG490 was also clearly seen by light microscopic measurement (Fig. 6B, lower panel). AG490 dramatically reduced intracellular TG contents in a concentration-dependent manner (Fig. 6C). In addition, AG490 concentration-dependently inhibited STAT-3 phosphorylation induced by 2 h treatment with MDI, suggesting the drug's efficacy (Fig. 6D). Fig. 6E is the densitometry result of Fig. 6D.

### KMU-3 inhibits the mRNA expressions of FAS, leptin, RBP4, and RANTES during adipocyte differentiation

**Figure 7:**
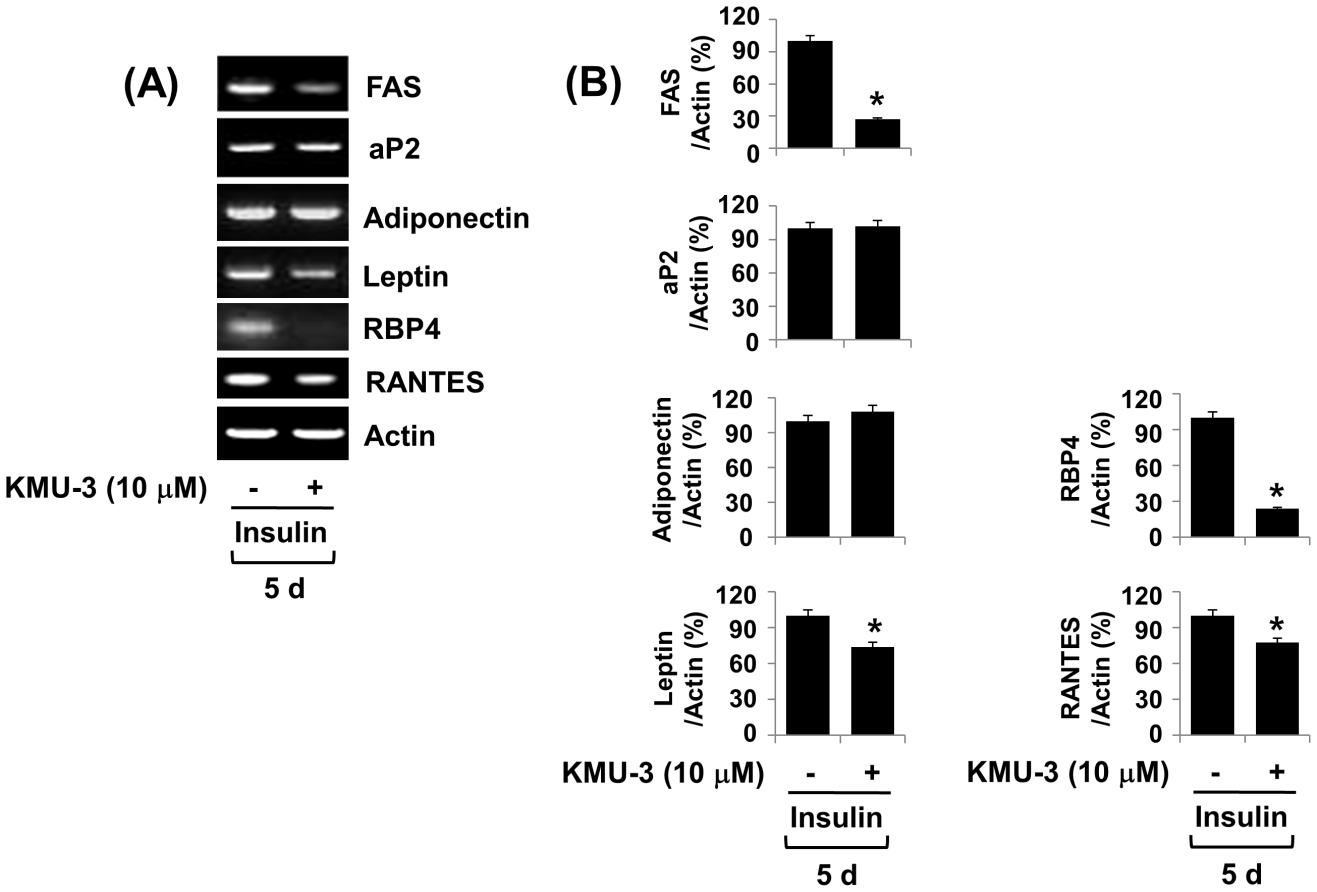
Effect of KMU-3 on mRNA expressions of adipcoyte-specific genes and adipokines during 3T3-L1 adipocyte differentiation. (A) 3T3-L1 preadipocytes were induced to differentiate with induction medium containing MDI, insulin, and FBS in the presence or absence of KMU-3 (10 µM), and harvested at day 2, 5, and 8, respectively. The cellular mRNA at each time point was extracted and analyzed by RT-PCR analysis. Each picture is a representative of three independent experiments. (B) The densitometry of (A) in which mRNA expression levels of FAS, aP2, adiponectin, leptin, RBP-4, and RANTES were quantified with those with action, respectively. *p<0.05 compared to the value obtained at the same time point (day 5) in the absence of KMU-3.

We next investigated whether KMU-3 (10 µM) regulates the expression of adipocyte-specific genes and/or adipokines during adipocyte differentiation. As shown in Fig. 7, results of RT-PCR assays showed a time-dependent increase in the mRNA expressions of FAS, aP2, adiponectin, leptin, RBP4, and RANTES in 3T3-L1 preadipocytes incubated with induction medium containing insulin on day 5. Notably, KMU-3 blocked the insulin-induced mRNA expression of FAS, but not aP2. However, while KMU-3 had no effect on the insulin-induced adiponectin mRNA expression, it slightly reduced the mRNA expression of leptin and RANTES induced by insulin. KMU-3 also had an ability to strongly inhibit the insulin-induced mRNA expression of RBP4. Control actin mRNA expression remained constant under these experimental conditions. The densitometry result of Fig. 7A is shown in Fig. 7B.

## Discussion

Excessive adipocyte differentiation confers abnormal expansion/accumulation of adipose tissue leading to high secretion of pathological adipokines, which are implicated in inflammation, insulin resistance, and metabolic disorders. Thus, potent inhibitors of adipocyte differentiation may have therapeutic potential as anti-obesity drugs. Based on this concept, here, we screened 53 compounds derived from a number of polyphenolic natural compounds and showed that among them, KMU-3, a novel derivative of gallic acid (GA), showed strong anti-adipogenic effect. We further demonstrated that this effect is mediated through modulation of the expression of C/EBP-α and PPAR-γ and the phosphorylation of STAT-3.

Mature or differentiated adipocyte has a spherical shape and is filled with many lipid droplets, which is distinguished from fibroblast-like preadipocyte in morphology [22]. We have demonstrated in this study that KMU-3, especially at the dose of 10 µM largely inhibits lipid accumulation without inducing cell toxicity during 3T3-L1 adipocytes differentiation of 8 days (Fig. 2, 3A and 3C), stressing its strong anti-adipogenic effect. Data of MTS here further show that treatment with KMU-3 (10 µM) for 24 h is also not cytotoxic to 3T3-L1 preadipocytes (Fig. 1). In contrast, treatment with GA, the parent compound of KMU-3, has no inhibitory effect on adipogenesis and cell growth during adipocyte differentiation up to 90 µM concentration (Fig. 3B), suggesting that GA up to 90 µM lacks anti-adipogenic activity. Aforementioned, GA at high concentrations (50–250 µM) has already been shown to induce apoptosis in 3T3-L1 preadipocytes (20,21). The differences in the finding between the current study and previous studies in the inhibitory effect of GA on growth of 3T3-L1 adipocytes may be due to experimental systems that are applied under the different condition (treatment time (1–3 days vs 8 days) or differentiation condition (no differentiation vs induction of differentiation). Earlier findings showed that transepithelial transport rate of GA (the value of ClogP: 0.43) is less than 1% for 40 min [23] while transepithelial transport rate of propyl GA (ClogP: 1.99) is about 50% for 60 min, largely due to increased lipophilicity [24]. Given that KMU-3 has high lipophilicity (ClogP: 3.02), which was achieved through attachment of hydrophobic 4-*tert*-butylaniline group by amide linkage, it is obvious that KMU-3 is more lipophilic and thus more readily cell membrane permeable than GA. Together, it is conceivable that the differential effect of KMU-3 vs GA on lipid accumulation in the preadipocytes is likely due, at least in part, to their difference in cell membrane permeability. Our study supports that the diversity-oriented synthesis strategy is an effective tool to generate a novel molecule KMU-3 with anti-adipogenic property from the material such as GA that lacks anti-adipogenic activity.

Aforementioned, adipocyte differentiation and maturation is largely influenced by the expressions and/or activity of the family of C/EBPs and PPARs [9]–[11], [25], [26], as evidenced by the fact that knocking out C/EBPs or PPAR-γ decreases or impairs white adipose tissue in mice [27]–[29]. Accumulating evidence also suggests that C/EBP-β and C/EBP-δ are induced in the early stages of adipogenesis [9], [23], [28], which subsequently up-regulates the expression of C/EBP-α and PPAR-γ [30], [31]. In this study, KMU-3 largely blocks the expressions of C/EBP-α and PPAR-γ, but not C/EBP-β, during adipocyte differentiation (Fig. 4A–D), suggesting that the KMU-3-mediated anti-adipogenic effect is largely due to the reduced expression of C/EBP-α and PPAR-γ and the KMU-3′s repressive effect on C/EBP-α and PPAR-γ expressions is independent of C/EBP-β. Recent findings also have shown that increased expressions and/or activities of C/EBPs and PPARs are necessary for the expression of adipocyte-specific genes and adipokines, including FAS, aP2, leptin, adiponectin, RBP4, and RANTES [32]–[34]. Among those, FAS is a lipogenic enzyme involved in fatty acid synthesis and its expression is largely increased in cells or tissues with high rates of fatty acid synthesis [35]. aP2 is a carrier protein for long chain fatty acids [36]. It was previously shown that once 3T3-L1 preadipocytes are differentiated with dexamethasone and isobutylxanthine, the preadipocytes acquire the characteristics of bona fide fat cells including responsiveness to insulin and induction of FAS and aP2 [37]. Based upon their findings, we also demonstrate that 3T3-L1 cells treated with insulin that had primarily been induced to undergo adipocyte differentiation by treatment with MDI largely induce the mRNA expressions of FAS and aP2 (Fig. 7A and B). However, KMU-3 inhibits the insulin-induced mRNA expression of FAS, while not affecting mRNA expression of aP2. These results suggest that KMU-3 has anti-lipogenic effect by repressing FAS expression, which may further contribute to the KMU-3-mediated anti-adipogenesis. It was proposed that the maturation of adipocyte differentiation is characterized by the capacity of the cells to synthesize and secrete some of adipokines, which are involved in the endocrine control of energy homeostasis [38], [39]. Thus, considering the present findings that KMU-3 reduces the mRNA expressions of leptin, RBP4, and RANTES during adipocyte differentiation process, KMU-3 may further repress adipocyte differentiation maturation process. A large body of literature has reported that adiponectin plays a positive role in control of obesity [40] and its overexpression enhances insulin sensitivity in part through effects on hepatic glucose production [41]–[43], whereas abnormal expression/secretion of some of adipokines, such as leptin, RBP4, and RANTES, is linked to obesity and obesity-related disease [44]–[46]. Therefore, specific small molecule inhibitors such as KMU-3 identified in this study are useful leads for future drug development efforts for prevention and/or treatment of obesity and/or obesity-related disease in which overexpression of leptin, RBP4, and/or RANTES plays pathological roles.

It has been shown that the family of STATs, including STAT-1, STAT-3, STAT-5, and STAT-6, is expressed in both 3T3-L1 preadipocytes and adipocytes [11], and several members of the STAT family, including STAT-1 and STAT-5, are critical for 3T3-L1 adipocyte differentiation [47], [48]. Interestingly, recent evidence further demonstrates that STAT-3 is rapidly activated in a few hours, and the active (phosphorylated) STAT-3 plays a critical role during the early stages of adipogenesis [12], [49]. In the current study, STAT-3 is also highly phosphorylated in 3T3-L1 preadipocytes treated with MDI at the early times (2 and 8 h) (Fig. 5A and D), lasting high levels on day 8 (Fig. 4C and F). A striking finding is the ability of KMU-3 to interfere with the MDI's, but neither insulin nor FBS, signaling to activate STAT-3 in 3T3-L1 cells (Fig. 4C, 4F, 5A, and 5D) and also to block the early adipogenesis process (Fig. 6A). Several non-receptor tyrosine kinases, including JAK2 and Src, are known to lie upstream of STAT-3 phosphorylation in response to extracellular stimuli [50]. However, assuming that the phosphorylation levels of STAT-1, STAT-5, JAK-1, JAK-2, Src, and ERK-1/2 remained largely unchanged, although they were expressed and phosphorylated in the MDI-treated cells, there seems to be no crosstalk between these signaling proteins (pathways) and STAT-3 phosphorylation in response to MDI exposure. Recent studies have reported that treatment with Stattic (a small molecule inhibitor of STAT-3) at 5 µM or AG490 (a JAK2/STAT-3 inhibitor) at 100 µM largely inhibits adipogenesis [49], [51]. Our experiments confirmed that treatment with AG490 at 100 µM greatly inhibits lipid accumulation (Fig. 6B and C) and the MDI-induced STAT-3 phosphorylation (Fig. 6D and E), strongly supporting that STAT-3 inhibition is crucial for the KMU-3-mediated anti-adipogenesis. Of note, STAT-3 regulates adipocyte differentiation via PPAR-γ [51] and STAT-3 activity is also necessary for the adipogenesis-induced expression of C/EBP-α and PPAR-γ [49], [51]. Thus, it is likely that KMU-3 may inhibit adipogenesis primarily through inhibition of STAT-3 at the early adipogenesis process, which subsequently down-regulates the expression of C/EBP-α and PPAR-γ. Previous studies thus far have not fully demonstrated which component(s) among MDI mediates STAT-3 phosphorylation in 3T3-L1 cells. In this study, we have clearly shown that IBMX, but not dexamethasone and insulin, is responsible for STAT-3 phosphorylation in the preadipocytes (Fig. 5B and E) and KMU-3 largely blocks it (Fig. 5C and F). IBMX which is a phosphodiesterase inhibitor has been shown to increase intracellular amounts of calcium and cAMP leading to activation of PKA, which subsequently phosphorylates cAMP-responsive element binding protein (CREB) [52]. There is also a previous report addressing PKA-dependent STAT-3 activation [53]. We demonstrate that treatment with H89 (PKA inhibitor) substantially inhibits the IBMX-induced STAT-3 phosphorylation and combination treatment with KMU-3 and H89 almost completely blocks it (data not shown). Together, our findings suggest that in the preadipocytes IBMX may rapidly trigger activation of calcium/cAMP/PKA signals, which subsequently activates STAT-3, and KMU-3 may inhibit STAT-3 phosphorylation via interference of the IBMX-induced calcium/cAMP/PKA signals in the early adipogenesis process.

Oxidative stress is highly increased in obese adipose tissue and participates in obesity and metabolic syndrome [54]. It has been shown that anti-oxidants such as α-lipoic acid and genistein are effective against obesity [55], [56]. These findings suggest that oxidative stress is linked to obesity. As mentioned before, KMU-3 is a synthetic derivative of GA, a naturally abundant plant polyphenolic compound with anti-oxidative property. Using DPPH-based anti-oxidative assay in cell-free system, we herein have found that the anti-oxidative activity (the value of RC_50_) of KMU-3 is 5.01 µM, which is similar to that of (5.52 µM) of Trolox (6-hydroxy-2,5,7,8-tetramethylchroman-2-carboxylic acid), a known anti-oxidant (data not shown). Given that strong anti-adipogenic effect is induced by KMU-3 at the doses of 5 and 10 µM (Fig. 2 and 3A), the KMU-3′s anti-adipogenesis effect is likely to be further in part attributed to its anti-oxidative activity. SHP-1, a protein tyrosine phosphatase, has been shown to dephosphorylate many target proteins including STAT-3 [57]. Of note, it has recently been shown that butein, a plant polyphenolic compound with anti-oxidative property, inhibits adipogenesis and STAT-3 phosphorylation in 3T3-L1 cells through induction of SHP-1 [58]. However, we observed that the SHP-1 protein expression is not altered in the preadipocytes treated with MDI in the presence or absence of KMU-3 (Fig. 5A and D), implying that although KMU-3 has anti-oxidative property, its inhibitory effects on adipogenesis and STAT-3 phosphorylation are independent of SHP-1.

Collectively, we report for the first time that KMU-3 has a strong anti-adipogenic effect and provide evidence that the effect is largely mediated through down-regulation of STAT-3, PPAR-γ, C/EBP-α, and FAS. Although there are still important issues that remain to be resolved, including the anti-adipogenic effect of KMU-3 on obese animal models, our findings presented here suggest that KMU-3 may be a promising novel compound for the treatment of obesity and related metabolic disorders.
